# Position-Specific Kinanthropometric Traits of Professional American Football Players: A Study of Mexican LFA Players

**DOI:** 10.3390/jfmk11010109

**Published:** 2026-03-05

**Authors:** Luis Gerardo Vázquez-Villarreal, Wiliam Carvajal-Veitía, Gustavo Guevara-Balcázar, Claudia Maceroni, Pedro López-Sánchez, María del Carmen Castillo-Hernández

**Affiliations:** 1 Medical Direction & Applied Sports Sciences, LFA Professional American Football League, Mexico City 01219, Mexico; gerardo.vazquez@lfa.mx; 2 Cardiovascular Pharmacology and Experimental Hyperbaric Medicine Laboratory, Postgraduate and Research Section, Superior School of Medicine, National Polytechnic Institute, Mexico City 11340, Mexico; gguevarab@ipn.mx; 3 Subdirectorate of Teaching and Research, Institute of Sports Medicine (IMD), Havana 10800, Cuba; 4 Ibero-American Network of Researchers in Applied Anthropometry (RIBA2), 04120 Almería, Spain; 5 Sports Science Research, Bellevue, WA 98008, USA; claudia@threebalance.com; 6 Postgraduate and Research Section, Superior School of Medicine, National Polytechnic Institute, Mexico City 11340, Mexico; elopezs@esm.ipn.mx; 7 Chronic Degenerative Disease Laboratory, Postgraduate and Research Section, Superior School of Medicine, National Polytechnic Institute, Mexico City 11340, Mexico; ccastillohe@ipn.mx

**Keywords:** American football, kinanthropometry, anthropometry, body composition, somatotype

## Abstract

**Background**: This cross-sectional observational study aimed to describe the position-specific kinanthropometric characteristics of Mexican professional American football players competing in the 2019–2020 seasons of the Liga de Fútbol Americano. **Methods**: A total of 189 athletes were assessed following International Society for the Advancement of Kinanthropometry standards. Twenty-six anthropometric variables were measured to estimate body composition (five-way fractionation), somatotype, proportionality indices, and tissue-specific masses. Positional differences were examined using ANOVA or Kruskal–Wallis tests with corresponding effect sizes (η^2^ or ε^2^). An exploratory stepwise discriminant analysis identified the anthropometric dimensions contributing most to positional differentiation, and classification accuracy was calculated. **Results**: Offensive and defensive linemen showed the greatest absolute size and higher adipose, muscle, and bone mass compared with other positions. The overall somatotype corresponded to a balanced endomorphic mesomorph (3.8–7.0–0.8), with wide receivers and defensive backs presenting lower endomorphy. The discriminant model identified arm relaxed girth, biiliocristal breadth, and sitting height as the variables contributing most to positional differentiation, achieving a classification accuracy of 57.7%. Given its exploratory nature and the absence of cross-validation, the discriminatory capacity of the model should be interpreted with caution. Somatotype Attitudinal Mean indicated greater interpositional heterogeneity among linemen. **Conclusions**: This study provides population-specific reference data for Mexican professional American football players, highlighting clear positional morphological characteristics. These findings may support talent identification and positional profiling; however, the exploratory discriminant model and league-specific sample limit generalization to other populations.

## 1. Introduction

American football is one of the most influential sports of the past century. Its popularity has expanded beyond North America, with notable growth in Europe and Asia [1,2,3,4]. This global expansion has sparked a surge in scientific interest in the physical characteristics of American football players, particularly within the fields of applied sports science and public health.

Traditionally, research on American football players has focused on orthopedics, neurology, and epidemiology, addressing topics such as traumatic injuries, concussions, chronic disease prevalence, and methodological concerns related to body composition assessment [5,6,7,8,9,10,11,12,13]. While performance in American football depends on a combination of stature, speed, and power, several studies have demonstrated that anthropometric traits are closely linked to physical performance demands [14,15,16,17,18,19,20,21,22,23,24,25,26,27]. These findings emphasize the importance of morphological profiling in understanding positional requirements.

Research into anthropometry at collegiate and professional levels has documented substantial changes in player morphology over time, including increases in body mass and stature, particularly among linemen [28,29,30]. However, despite the global expansion of the sport, Latin American athletes remain underrepresented in the literature. Mexican players constitute a distinct population with a long-standing competitive tradition; however, their morphological characteristics have not been systematically documented [31,32].

While the anthropometric profiles of players from North America, Europe and Asia have been extensively described, there is a notable lack of research on Latin American professional athletes. Understanding how Mexican players compare with international profiles is essential for improving talent identification, positional development, and evidence-based recruitment strategies in the region.

Although previous studies have employed advanced methods such as hydrostatic weighing, air-displacement plethysmography, bioelectrical impedance, and dual-energy X-ray absorptiometry (DXA) [9,13,14,18,20,21,33], kinanthropometric approaches based on ISAK standards provide additional benefits. These methods provide a broader set of morphological variables, including segmental lengths, girths, breadths and proportionality indices, which are not captured by imaging and densitometric techniques due to their measurement principles. This wider range of variables enables a more detailed examination of positional differences and may reveal morphological dimensions that remain undetected by laboratory-based methods. Furthermore, identifying which anthropometric traits contribute most to positional discrimination can generate insights that go beyond traditional body composition metrics, supporting the development of future hypotheses regarding potential associations between morphological characteristics and sport-specific performance.

We hypothesized that professional players from the Mexican LFA would exhibit population-specific, position-related morphological differentiation, reflected in distinct anthropometric, body composition, somatotype and proportionality profiles. Additionally, we expected that a subset of absolute anthropometric dimensions would show exploratory discriminatory potential for distinguishing playing positions, although with limited classificatory accuracy given the multivariate variability of this population.

## 2. Materials and Methods

### 2.1. Study Design

A cross-sectional descriptive observational study was conducted to develop the kinanthropometric profile of Mexican professional American football players participating in the 2019–2020 regular seasons of the Professional American Football League (Liga de Fútbol Americano, LFA). The study followed the Strengthening the Reporting of Observational Studies in Epidemiology (STROBE) guidelines [34].

### 2.2. Setting

Anthropometric assessments were performed during the competitive stage of the season, with measurement sessions scheduled in coordination with the medical staff of each team. All evaluations were conducted in medical facilities located within professional training centers, including locker rooms, consultation rooms and therapy areas. These indoor environments maintained controlled temperature and humidity conditions to ensure measurement consistency.

Data collection took place in February and March, during the winter season in Mexico. Although this period is characterized by cooler temperatures, no extreme climatic variations were observed across the measurement sites, which included Mexico City, Estado de México, Monterrey and Coahuila. The stable environmental conditions minimized external variability and contributed to consistent measurement reliability throughout the study.

### 2.3. Participants, Study Size and Eligibility Criteria

A total of 189 professional athletes participated in the study, distributed across seven playing positions: defensive backs (DB, *n* = 30), defensive linemen (DL, *n* = 33), linebackers (LB, *n* = 33), offensive linemen (OL, *n* = 27), quarterbacks (QB, *n* = 18), running backs (RB, *n* = 16) and wide receivers (WR, *n* = 31). These 189 players represented 51.38% of all Mexican athletes registered in the Liga de Fútbol Americano (LFA) during the 2019–2020 seasons, providing substantial coverage of the league’s national player population.

The mean chronological age of the sample was 28.1 years (range: 21.3–40.0 years). Players also demonstrated extensive competitive development, with a mean sporting age of 12.1 years (range: 7–20 years), reflecting long-term training exposure and positional specialization. Recruitment was conducted in collaboration with team medical staff to ensure standardized assessment conditions within professional training environments.

Inclusion criteria were:

- Mexican nationality and official registration in the LFA;
- a minimum of five years of competitive experience;
- age between 21 and 40 years;
- absence of musculoskeletal injuries in the previous six months;
- ability to complete all anthropometric assessments.

Exclusion criteria were:

- foreign athletes—primarily North American players who participate in the LFA as international reinforcements;
- uncontrolled chronic diseases;
- recent surgery or physical impairments that could compromise measurement accuracy;
- acute illness at the time of evaluation;
- non-compliance with pre-assessment procedures (e.g., scheduling, hydration protocols).

All participants provided written informed consent. The study was conducted in accordance with the Declaration of Helsinki [35] and approved by the Ethics Committee of the Liga de Fútbol Americano (Approval code: 01-2019).

### 2.4. Anthropometric Assessment

A total of 26 anthropometric indicators were assessed, including body mass, stretch stature, sitting height, arm span, six breadths, eight girths and eight skinfolds, following the standardized procedures of the International Society for the Advancement of Kinanthropometry (ISAK) [36]. From these measurements, indirect indicators of body composition, somatotype, proportionality and additional anthropometric indices were derived as described below.

All measurements included in the database were obtained by three ISAK Level 3 anthropometrists, each of whom is an accredited ISAK Instructor, ensuring the highest level of technical proficiency and adherence to international standards. The equipment used included a digital scale accurate to 0.1 kg (Omron Healthcare Co., Ltd., Kyoto, Japan); a stadiometer accurate to 0.1 mm (SmartMet S.A. de C.V., Guadalajara, Jalisco, Mexico); a Harpenden skinfold caliper (10 g/mm^2^) accurate to 0.2 mm (Harpenden, British Indicators, Crymych, UK); small and large sliding calipers accurate to 1 mm (SmartMet S.A. de C.V., Guadalajara, Jalisco, Mexico) for bone breadths; and a flexible Lufkin W606PMP anthropometric tape accurate to 1 mm (Apex Tool Group, LLC, Sparks, MD, USA) was used for girth measurements.

Body composition was determined using the anthropometric fractionation method of Ross and Kerr [37]. Fractions of adipose, muscle, skeletal, residual and skin mass were obtained based on the 25 anthropometric dimensions measured. Absolute (kg) and relative (%) values were calculated for all tissues, with relative values expressed as: [estimated tissue mass (kg) × 100]/body mass. The difference between body mass obtained by scale and that predicted by the Ross and Kerr method was expressed in absolute (kg) and relative (%) terms and defined as: [mass obtained by scale − estimated mass per Ross and Kerr method] × 100/mass obtained by scale.

Although the Ross and Kerr anthropometric fractionation method has been validated in both athletic and non-athletic populations, the authors have noted that the accuracy of anthropometric techniques can vary depending on the morphological characteristics of the population in question [37]. For this reason, and as part of the methodological process aimed at ensuring the precision of the estimates in this specific sample, an internal concordance verification was conducted prior to the method’s analytical application. This involved comparing scale-measured body mass with the sum of the five estimated masses, and assessing the degree of agreement between them using standard reliability statistics.

The results of this verification are presented in the Section 3. Somatotype was determined using the Heath–Carter anthropometric method [38], which classifies physique into endomorphy, mesomorphy and ectomorphy. Component values were categorized as low (0–2.9), moderate (3–5), high (5.5–7) or very high (>7). The dominant component defined the somatotype category for each playing position; differences smaller than 0.5 units were considered negligible.

Somatotype Attitudinal Mean (SAM) was calculated using the average somatotype of the entire sample (*n* = 189) as the reference, following the procedure described by Heath and Carter [38]. Individual deviations from the group mean were summed and divided by the number of players in each position. The magnitude of deviation for each position relative to the total sample was illustrated using box-and-whisker plots. Mean somatotypes for each position were also plotted as x:y somatopoints on a somatochart according to the Heath–Carter method.

As a measure of body proportionality, phantom Z-scores were obtained for adipose, muscle, skeletal, and residual tissue masses [37]. The calculated indices include:

- Body Mass Index (BMI) (kg/m^2^), representing the relationship between body mass and stretched stature.
- Σ6SF The sum of six skinfold thicknesses (triceps, subscapular, supraspinale, abdominal, thigh, and calf), expressed in millimeters (mm).
- Σ8SF The sum of eight skinfold thicknesses (triceps, subscapular, biceps, iliac crest, supraspinale, abdominal, thigh, and calf) expressed in millimeters (mm).
- Muscle to bone ratio, expressed as the relationship between muscle and bone mass in kilograms [39].
- Arm span to stretched stature ratio (Ape Index), providing an estimation of relative arm span, calculated as arm span/stretched stature [40].
- Cormic Index, estimating relative trunk length, calculated as sitting height/stretched stature [40].
- Relative biacromial breadth, indicating shoulder proportionality, calculated as biacromial breadth/stretched stature [40].
- Relative biiliocristal breadth, representing pelvic proportionality, calculated as biiliocristal breadth/stretched stature [40].
- Shoulder-Hip index, describing the torso shape to analyze biomechanical advantages for stability and strength, calculated as biacromial breadth/biiliocristal breadth [40].
- Manouvrier Index, providing a relative estimation of lower limb length, calculated as the ratio of the difference between stretched stature and sitting height to stretched stature [40].
- Locomotive Index, describing the relationship between load and musculoskeletal tissues to analyze locomotor system efficiency, calculated as the ratio of the sum of adipose and residual tissues to the sum of muscle and bone mass [41].

### 2.5. Biases

Several methodological strategies were implemented to minimize bias and enhance the reliability of the study. Selection bias was reduced by applying strict eligibility criteria to ensure a homogeneous sample of Mexican professional athletes with comparable competitive experience. Foreign players, primarily North American athletes, were excluded to avoid heterogeneity related to different training backgrounds.

Measurement bias was minimized by performing all assessments under standardized indoor conditions and using calibrated equipment. Although three ISAK Level 3 anthropometrists participated in the study, all measurements included in the database were obtained by a single ISAK Level 3 Instructor, eliminating inter observer variability. Intra observer technical error remained below 5% for skinfolds and below 1% for all other measurements, consistent with ISAK standards.

To ensure data reliability, the Ross and Kerr anthropometric fractionation method was subjected to an internal validation procedure within this sample, confirming high agreement between measured and estimated body mass and minimizing systematic error.

Potential confounding factors were controlled by evaluating players under consistent pre assessment conditions, reducing the influence of hydration status, fatigue and recent dietary intake on body composition outcomes. These methodological precautions strengthened the accuracy and validity of the study.

### 2.6. Statistical Analysis

Descriptive statistics were calculated using the mean ($\bar{X}$) and standard deviation (SD). The reliability of the body composition assessments was examined using Spearman’s correlation, R^2^, residual standard error (RSE), 95% confidence intervals for correlations and intraclass correlation coefficients (ICC).

One-way ANOVA was used to test for differences among the seven playing positions for variables that met the assumptions of normality and homoscedasticity (Kolmogorov–Smirnov or Shapiro–Wilk test; Levene’s test). When the assumption of homogeneity of variances was violated, Welch’s ANOVA was applied. When the normality assumption was not met, the Kruskal–Wallis test was used. When global significance was detected, Games–Howell post hoc tests were applied for ANOVA/Welch contrasts, and Dunn post hoc tests were applied for Kruskal–Wallis. These adjusted procedures were selected to control for type I error inflation associated with multiple comparisons and to identify specific positional differences.

The effect size (f) obtained from the ANOVA was converted to partial eta squared (η^2^). For the Kruskal–Wallis test, epsilon squared (ε^2^) was calculated. Statistical power ranged from 0.95 to 0.96 for all evaluated variables, indicating a high probability of detecting real effects and minimizing the risk of type II errors [42].

As the players were organized into four different teams, the potential cluster effect related to team-based training environments was considered. However, no multilevel or hierarchical modeling was applied, since the objective of the study was to create descriptive profiles rather than to make inferences across clusters.

Due to the large number of variables included in the study (26 absolute anthropometric dimensions and 19 derived indicators relating to body composition, somatotype and proportionality), a multivariate approach was necessary to prevent the inefficiency and increased type I error associated with carrying out numerous univariate tests. For this reason, discriminant analysis was applied for exploratory purposes only, to identify general trends and the subset of absolute anthropometric dimensions with the greatest combined discriminatory capacity across playing positions.

An exploratory stepwise discriminant analysis was then conducted to evaluate the discriminatory capacity of the anthropometric variables. Only the variables that met the univariate normality criteria were included: stretch stature, sitting height, arm span, biacromial breadth, arm relaxed girth, thigh girth, abdominal skinfold and triceps skinfold. Homogeneity of covariance matrices was confirmed using Box’s M test (*p* = 0.092) and multicollinearity was assessed using VIF < 5. Linearity and independence of observations were verified. As multivariate normality could not be fully guaranteed, the discriminant analysis was interpreted cautiously and considered exploratory.

A territorial map was constructed to visualize positional differences, and classification accuracy was reported for each position. he significance level was set at *p* < 0.05. Analyses were performed using Statistica 9.0 (StatSoft Inc., Tulsa, OK, USA), IBM SPSS Statistics 25.0 (IBM Corp., Armonk, NY, USA), and MedCalc version 23.2.1 (MedCalc Software Ltd., Ostend, Belgium).

## 3. Results

### 3.1. Descriptive Data

#### 3.1.1. Anthropometric Attributes by Playing Position

From a descriptive standpoint, there were marked differences in anthropometric characteristics across playing positions. Offensive and defensive linemen displayed the greatest absolute size, with higher values for body mass, stretch stature, sitting height, breadths, and limb girths. These positions also exhibited the highest subcutaneous adiposity, particularly in the subscapular, front-thigh, and calf skinfolds. In contrast, skill-position players (wide receivers, running backs, and defensive backs) showed lower overall mass and smaller breadth and girth dimensions. Detailed descriptive values for all variables are presented in Table 1.

#### 3.1.2. Body Composition, Somatotype and Indices

For this sample, the reliability of the five-way fractionation method is evidenced by the comparison of body mass values from a scale (97.1 ± 19.3 kg) and those derived from the sum of the five masses (102.1 ± 23.4 kg). Statistical tests showed a Spearman correlation coefficient of 0.985 (*p* < 0.001; 95% CI = 0.9804–0.9889), an R-squared value of 0.971 (*p* < 0.001; 95% CI = 0.961–0.977), an RSE = 3.2 kg, and an intraclass correlation coefficient of 0.983 (*p* < 0.001; 95% CI = 0.978–0.988), indicating high reliability and agreement of the method used.

From a descriptive perspective, clear positional differences were observed in body composition. Offensive and defensive linemen exhibited the greatest absolute and relative adipose tissue mass, whereas defensive backs and wide receivers showed the lowest proportional adiposity, reflected in markedly negative Z-scores. Linemen also presented the highest BMI values, consistent with their elevated muscle-to-bone ratio and larger structural dimensions (Table 2).

The overall somatotype of the sample corresponded to an endomorphic–mesomorph profile. At the descriptive level, offensive and defensive linemen displayed the most pronounced endomorphic–mesomorphic characteristics, with high endomorphy and very high mesomorphy, while wide receivers and defensive backs showed substantially lower endomorphy. Linebackers, offensive linemen, and defensive linemen demonstrated the most extreme mesomorphic development.

Anthropometric indices also showed descriptive differences across positions. Linemen presented the highest shoulder-hip and locomotive indices, consistent with their greater mass and breadth dimensions, whereas skill-position players exhibited lower values across these structural indicators. Detailed values for all components and indices are provided in Table 2.

### 3.2. Main Results

#### 3.2.1. Anthropometric Markers for Playing Position Classification

From an exploratory multivariate perspective, the stepwise discriminant function analysis identified two significant functions capable of differentiating playing positions. Wilks’ lambda for the overall model was λ = 0.228 (χ^2^ = 265.8, *p* < 0.001), indicating that the retained variables contributed meaningfully to positional discrimination. Discriminant Function 1 (DF1) accounted for 85.4% of the explained variance (eigenvalue = 2.116; canonical correlation = 0.824), whereas Discriminant Function 2 (DF2) explained an additional 7.7% (eigenvalue = 1.190; canonical correlation = 0.399).

Of the 26 anthropometric variables initially entered, only arm relaxed girth, biiliocristal breadth, and sitting height contributed significantly to the discriminant model. DF1 was driven primarily by arm relaxed girth (standardized coefficient = 0.889) and biiliocristal breadth (0.743), with sitting height contributing to a lesser extent (0.316). DF2 was defined mainly by stretch stature (0.741) and abdominal skinfold thickness (0.658).

The model correctly classified 57.7% of players (109/189). Offensive linemen showed the highest classification accuracy (85.7%), followed by quarterbacks (76.5%) and defensive linemen (60.6%). Linebackers were correctly classified in 54.5% of cases. Skill-position players showed greater morphological overlap, with lower classification rates: wide receivers (51.6%), defensive backs (38.7%), and running backs (37.5%).

Group centroids further illustrated positional separation. Along DF1, offensive linemen (2.434) and defensive linemen (1.486) were clearly separated from quarterbacks (−1.225), defensive backs (−1.156), running backs (−1.048), and wide receivers (−0.923). Linebackers (0.794) occupied an intermediate position. Along DF2, quarterbacks (1.013) and running backs (0.667) scored higher, whereas defensive linemen (−0.371) scored lower, reflecting differences in stretch stature and abdominal adiposity.

**Figure 1:**
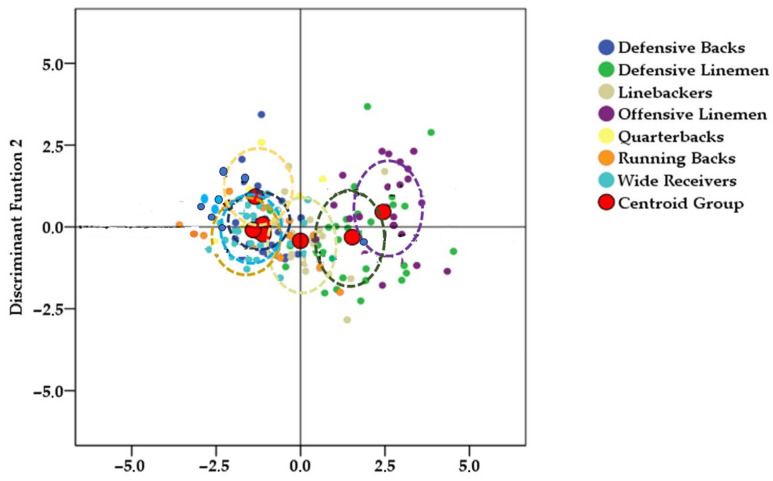
Territorial map of professional American football players in Mexico by playing position. The dotted circle around each centroid represents the spatial distribution of each position within the radius it occupies. Horizontal and vertical axes show individual discriminant scores for discriminant functions 1 (horizontal) and 2 (vertical), which reflect the distance of each position’s centroid along the respective analytic axis. Player positions are identified exclusively through the legend on the right side of the figure.

The territorial map (Figure 1) visually summarizes these patterns. Centroids represent the average discriminant position for each role, and the surrounding dashed circles illustrate within-group variability. Overall, DF1 provided the clearest separation—primarily distinguishing linemen from skill-position players, while DF2 contributed secondary distinctions related to stature and central adiposity.

#### 3.2.2. Body Composition and Anthropometric Index Comparison by Playing Position

Table 3 shows the results of the ANOVA and Kruskal–Wallis tests applied to body composition indicators and anthropometric indices across playing positions. The indices muscle-to-bone ratio, arm span, cormic index, biacromial breadth, and Manouvrier index did not show significant differences between positions (*p* > 0.05), with small effect sizes (η^2^ or ε^2^).

In contrast, several variables demonstrated significant differences (*p* < 0.05), particularly among offensive linemen, who consistently exhibited the highest values across multiple anthropometric characteristics. Post hoc comparisons (Games–Howell test for; Dunn test for Kruskal–Wallis) confirmed that offensive linemen differed significantly from most other groups in body mass (η^2^ = 0.621; large effect) and stretch stature (η^2^ = 0.297; large effect). Significant effects were also observed for adipose mass (η^2^ = 0.581; large effect) and its percentage (η^2^ = 0.297; large effect), as well as for muscle mass in both absolute (η^2^ = 0.545; large effect) and relative terms (η^2^ = 0.638; large effect).

Offensive linemen also showed significantly higher skeletal mass (η^2^ = 0.638; large effect) and residual mass (absolute: η^2^ = 0.571; large effect; percentage: η^2^ = 0.188; medium effect) compared with other positions. Additional variables with significant positional differences included skin mass (η^2^ = 0.595; large effect), BMI (η^2^ = 0.577; large effect), and the sum of six skinfolds (η^2^ = 0.453; large effect).

Regarding anthropometric indices, offensive linemen presented higher biiliocristal breadth (η^2^ = 0.332; medium effect), shoulder–hip ratio (η^2^ = 0.035; small effect), and locomotor index (η^2^ = 0.309; medium effect). Although these indices showed smaller effects compared with body composition variables, they still contributed to positional differentiation.

### 3.3. Additional Results

#### Graphical Representation and Positional Comparison of Somatotype

**Figure 2:**
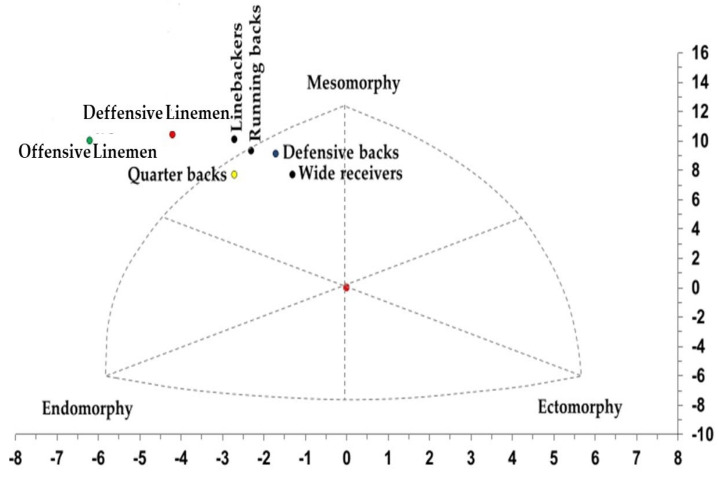
Somatotype distribution in Professional American Football players in México by Playing Position.

The somatotype chart (Figure 2) illustrates the mean somatotype position for each playing role. All groups clustered within the left region of the somatochart, reflecting a dominant mesomorphic component with endomorphy exceeding ectomorphy. Offensive and defensive linemen were positioned furthest from the remaining groups, indicating markedly higher mesomorphy and endomorphy, followed by linebackers. Skill-position players (wide receivers, defensive backs) occupied the lowest-endomorphy region of the chart.

**Figure 3:**
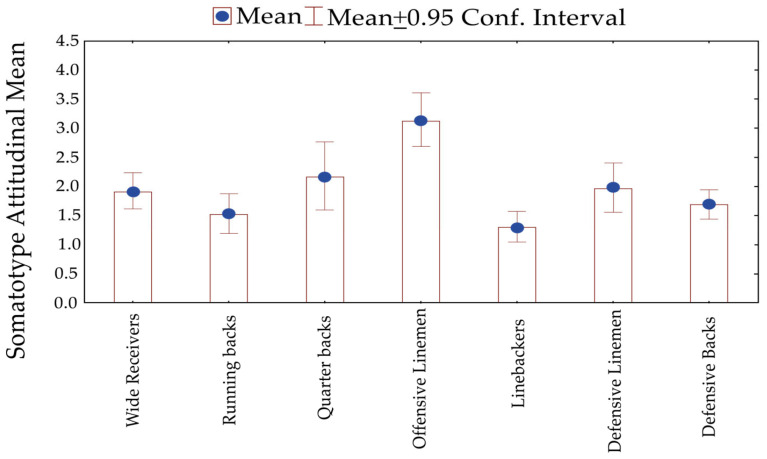
Somatotype attitudinal mean in Mexican Football players by playing position.

Figure 3 illustrates SAM’s descriptive characteristics. It is noteworthy that linebackers and defensive backs presented less 95% confidence interval in SAM than players in other positions. Wider 95%IC was found for quarterbacks, offensive and defensive linemen, represented in the figure by larger whiskers. The 95% confidence interval for offensive linemen overlapped only with that of quarterbacks, while showing no overlap with any other position. This indicates that, within the considered margin of error, the distribution of mean values for offensive linemen is relatively close to that of quarterbacks, while remaining distinctly separate from all other positions.

## 4. Discussion

### 4.1. Analysis of Absolute Size, Body Composition, and Somatotype

Clear positional differences were observed in the anthropometric profiles of professional American football players in Mexico. Linemen showed the greatest overall size, with higher body mass and larger skeletal and muscular dimensions, while skill-position players presented smaller breadths, girths, and lower adiposity. These tendencies are consistent with positional distinctions described in previous work across collegiate and professional levels [24,43].

The somatotype distribution indicated a predominantly endomorphic–mesomorphic profile, with linemen showing higher values for both components and skill-position players displaying lower endomorphy. Similar positional patterns have been reported in other football populations [38]. Although somatotype values in this sample fall within ranges described internationally, direct comparisons must be interpreted cautiously due to population-specific characteristics [44].

Studies of body composition—including the application of the Ross and Kerr anthropometric fractionation model and indices such as the muscle–bone index—have also contributed to highlighting positional morphological differences [45,46,47,48,49,50,51,52,53].

The discriminant analysis identified arm relaxed girth, biiliocristal breadth, and sitting height as the variables contributing most to positional separation. This model distinguished linemen from skill-position players, although the moderate classification accuracy reflects the overlap expected in multidimensional anthropometric traits. Comparable positional clustering has been described in previous analyses of elite football players [54].

The somatotype attitudinal mean showed greater variability among linemen and more homogeneous profiles among linebackers and defensive backs, a pattern consistent with descriptions of positional heterogeneity in team sports [4]. These observations illustrate the range of structural characteristics within and between positions, without implying functional implications.

Overall, the findings highlight consistent morphological distinctions across positions in this league and provide descriptive information for an understudied population of professional players in Mexico.

### 4.2. Analysis of Anthropometric Indices and Positional Specialization

The analysis of anthropometric indices showed structural differences across playing positions. Linemen presented higher values in indices related to pelvic breadth and locomotor structure, while wide receivers and running backs showed higher Manouvrier index values. These tendencies align with general descriptions of positional morphology in team sports and with previous observations in American football populations [55,56,57,58,59].

The discriminant analysis identified arm relaxed girth, biiliocristal breadth, and sitting height as the variables contributing most to positional separation. These dimensions reflect broad structural tendencies rather than functional implications, and the moderate classification accuracy indicates the expected overlap among multidimensional anthropometric traits [4].

Quarterbacks showed intermediate values across indices, consistent with descriptions of balanced morphological profiles in roles involving technical execution and decision-making [23]. Defensive backs and wide receivers exhibited lower shoulder-hip index values, a pattern previously associated with lighter body structures in positions requiring frequent movement and spatial adjustments [60].

Research in other sports has shown that body proportions may correspond to general biomechanical tendencies, such as force application, stability, or movement efficiency [40,61,62,63]. Although these findings provide contextual information, direct associations with performance in American football cannot be inferred from the present data.

Overall, the variability observed in anthropometric indices illustrates the range of structural characteristics across positions in this league. These indices offer descriptive information that may support morphological profiling, while acknowledging that further research is needed to clarify their relevance in performance-related contexts [64,65,66,67].

### 4.3. Limitations

This study presents several limitations. First, the sample did not include athletes from the kicker position, a role for which published anthropometric information remains scarce [4,20,21]. Second, body composition was assessed using anthropometric fractionation rather than two-compartment models such as DXA or air-displacement plethysmography, which are more commonly reported in studies of American football players [4,20,21].

The timing of data collection represents an additional limitation. Measurements were obtained before structural changes within the league and before the interruption caused by the COVID-19 pandemic, which restricts the ability to generalize the findings to current league conditions. Finally, the cross-sectional design does not allow examination of longitudinal changes or external validation of the observed patterns. Future studies incorporating repeated measurements and complementary assessment methods would strengthen the interpretation of morphological trends.

### 4.4. Generalization

The anthropometric profiles described in this study offer a reference for positional characteristics within this professional league. However, generalization beyond this context requires caution. Differences in training systems, competitive structures, and population characteristics may influence morphological patterns across leagues and countries [66,67,68,69]. Comparisons with other professional or collegiate programs could help identify shared tendencies and contextual variations, although methodological alignment—particularly in measurement protocols and body composition models—remains essential for meaningful cross-league interpretation [70].

Future research using standardized anthropometric procedures, such as ISAK-based protocols and proportionality indices, may contribute to broader comparative databases and support more consistent evaluations across competitive environments.

### 4.5. Relevance and Practical Applications

The present findings offer a descriptive framework for understanding positional morphology in professional American football players in Mexico. By using standardized anthropometric procedures, including ISAK-based measurements and proportionality indices, this study provides reference information that complements previous work relying primarily on imaging techniques such as hydrostatic weighing, air-displacement plethysmography, bioelectrical impedance, and DXA [13,14,18,20,21,24,44]. These anthropometric approaches allow for practical field-based assessments that can support routine monitoring in applied settings [68,69].

The use of body mass fractionation contributes additional detail to the characterization of structural components, expanding the descriptive tools available for evaluating athletes. Previous studies have reported the applicability of this method in team sports such as soccer and rugby [39,71,72], and the present results add information for its use in American football. Although anthropometric estimations differ conceptually from imaging-based models, they offer a practical alternative for profiling athletes in contexts where laboratory-based assessments are not feasible.

Overall, the anthropometric indices and fractionation-based measures presented here may assist practitioners in developing position-specific reference ranges and in monitoring morphological characteristics across competitive seasons. These applications remain descriptive, and further research is needed to clarify their relevance in performance-related contexts.

### 4.6. Future Research Directions

Future studies should incorporate complementary body composition methods, such as DXA or air-displacement plethysmography, to enable external validation of anthropometric estimations and to determine the most appropriate equations for this population, as has been done in other sports contexts [71,72,73]. Longitudinal designs would also help clarify how morphological characteristics evolve across competitive seasons and throughout playing careers, providing insight into age-related or training-related changes [14,17].

Research examining the relationship between anthropometric profiles and injury occurrence may offer additional value, particularly in positions with high mechanical demands [9,67,74]. Finally, the integration of advanced analytical approaches, including Big Data Technology and large datasets, may support the development of predictive tools for monitoring morphological trends and informing player evaluation processes [75].

## 5. Conclusions

This study described position-related morphological differences in professional American football players from the Mexican LFA. Distinct patterns were observed in anthropometric dimensions, body composition, somatotype, and proportionality indices, supporting the presence of population-specific structural variation across playing roles. The discriminant analysis identified a subset of absolute anthropometric variables with exploratory value for distinguishing positions, although the moderate classification accuracy reflects the expected overlap within this multivariate profile. Overall, the findings provide descriptive reference information for an understudied population and contribute baseline data for future research on positional morphology in American football.

## Figures and Tables

**Table 1:** Descriptive anthropometry of Professional American Football players in Mexico by playing position.

	DB		DL		LB		OL		QB		RB		WR		Total	
	X	SD	X	SD	X	SD	X	SD	X	SD	X	SD	X	SD	X	SD
Basic Variable																
Age	27.8	2.8	28.6	3.9	27.8	2.3	28.5	2.7	28.0	1.6	27.7	2.1	28.4	2.8	28.1	2.8
Body mass (kg)	83.4	6.6	111.3	16.2	93.4	7.4	127.6	16.8	86.0	11.0	84.1	10.8	83.2	7.9	97.1	19.8
Stretch stature (cm)	175.5	5.5	183.4	4.9	179.6	4.1	185.0	6.1	175.9	2.0	173.9	7.0	177.8	5.0	179.4	6.3
Sitting height (cm)	92.3	3.0	96.3	2.9	94.2	2.8	96.5	2.9	92.9	2.3	91.2	4.2	92.7	3.5	94.0	3.6
Arm span (cm)	179.9	6.8	188.8	5.7	183.0	6.0	189.5	6.2	180.1	2.1	178.4	7.1	180.7	5.6	183.6	7.3
Breadths (cm)																
Biacromial	40.7	1.9	43.5	1.9	42.0	1.6	44.1	2.4	40.6	1.7	41.2	2.1	41.2	1.6	42.1	2.3
Transverse chest	29.8	2.5	34.0	3.3	30.7	1.7	35.2	2.4	30.3	2.3	29.6	2.7	29.3	2.2	31.4	3.3
Antero-posterior chest depth	21.2	1.5	24.5	2.3	22.5	2.9	26.5	2.3	21.8	3.2	21.7	2.5	21.4	1.9	22.9	3.0
Biiliocristal	28.3	1.7	32.0	2.2	29.7	1.7	34.2	2.0	29.0	2.5	28.4	2.1	28.4	1.4	30.2	2.8
Humerus	7.0	0.4	7.6	0.4	7.4	0.4	7.8	0.5	7.3	0.2	7.0	0.4	7.0	0.4	7.3	0.5
Femur	10.0	0.3	10.8	0.7	10.3	0.6	11.3	0.9	10.1	0.7	10.0	0.5	9.9	0.5	10.4	0.8
Girths (cm)																
Head	56.3	2.4	57.5	1.5	57.5	1.0	58.8	2.0	56.4	1.4	56.0	1.4	57.1	2.0	57.2	2.0
Arm relaxed	35.2	1.5	40.4	2.8	37.6	2.1	42.3	2.6	34.8	2.3	35.6	2.8	34.5	1.6	37.6	3.6
Arm flexed and tensed	37.9	2.5	42.2	2.7	39.9	1.9	43.7	2.5	35.2	4.5	37.7	2.6	36.8	1.7	39.6	3.5
Forearm	29.1	1.2	31.7	2.5	30.7	1.2	34.2	5.8	29.3	1.5	29.1	1.5	29.1	1.4	30.6	3.2
Chest	102.6	4.5	117.0	7.7	107.1	5.1	125.7	7.9	102.8	5.5	103.1	7.9	101.9	4.7	109.5	10.6
Waist	86.4	4.6	101.2	9.6	91.3	5.1	112.3	8.1	89.9	8.7	88.5	7.0	85.9	6.1	94.1	11.6
Thigh 1 cm gluteal	62.5	2.4	70.3	5.3	65.2	2.4	74.3	3.9	62.6	4.8	61.8	3.9	61.5	3.0	66.0	5.9
Calf	37.7	2.6	41.7	2.9	39.6	2.2	43.5	3.8	37.8	3.0	37.5	2.8	37.6	1.8	39.6	3.5
Skinfolds (mm)																
Triceps	8.9	3.1	12.5	6.3	9.7	3.3	17.7	5.9	12.0	5.0	8.9	2.7	8.3	2.7	11.1	5.3
Subscapular	11.2	3.1	21.0	8.4	14.8	4.6	31.2	10.7	15.4	8.5	13.4	5.5	12.1	4.6	17.3	9.4
Biceps	4.0	1.2	6.6	3.1	4.6	1.4	9.5	3.7	5.9	2.4	4.9	1.7	3.9	1.2	5.6	3.0
Iliac crest	14.1	4.7	25.8	10.0	19.8	7.2	33.3	7.2	18.8	8.1	17.7	8.8	14.3	7.2	20.8	10.0
Supraspinale	7.6	2.5	17.0	8.3	11.3	4.9	26.0	6.2	11.5	6.3	9.9	5.4	8.1	4.2	13.2	8.2
Abdominal	18.8	7.8	29.9	9.7	23.5	7.6	36.5	7.0	23.0	10.8	21.1	8.1	18.8	8.4	24.7	10.3
Thigh	10.7	3.5	15.6	7.3	11.9	4.0	22.2	8.6	12.7	4.7	11.0	3.2	9.6	3.6	13.5	6.8
Calf	6.1	2.3	12.3	5.4	8.6	3.5	16.4	7.1	7.3	1.5	6.7	2.0	5.9	2.2	9.4	5.5

DB: defensive backs; DL: defensive linemen; LB: linebackers; OL: offensive linemen; QB: quarterbacks; RB: running backs; WR: wide receivers; X: mean; SD: standard deviation.

**Table 2:** Body composition, somatotype, and anthropometric indices in Professional American Football players in Mexico by playing position.

	DB		DL		LB		OL		QB		RB		WR		Total	
	X	SD	X	SD	X	SD	X	SD	X	SD	X	SD	X	SD	X	SD
Adipose mass																
kg	17.9	3.2	21.5	3.8	19.5	3.1	30.7	4.0	20.3	5.9	19.0	4.7	18.4	4.1	18.5	4.1
Z	−1.6	0.5	−1.4	0.4	−1.6	0.4	−1.6	0.6	−1.1	0.9	−1.4	0.6	−1.6	0.6	−0.6	0.5
%	21.5	3.5	21.0	2.3	21.0	3.0	24.1	1.4	24.5	4.2	22.4	314.3.6	22.0	3.5	20.9	2.7
Muscle mass																
kg	45.2	4.2	60.9	9.4	49.8	3.9	64.8	11.3	44.8	5.2	44.2	7.3	44.5	4.1	51.0	5.6
Z	3.1	0.6	3.7	2.9	3.2	0.8	5.0	1.1	3.0	0.8	3.3	0.9	2.7	0.5	3.0	0.8
%	54.2	2.7	54.7	2.4	53.8	3.0	50.8	3.6	52.2	2.8	53.5	3.6	53.6	2.7	52.5	2.3
Skeletal mass																
kg	8.8	0.9	9.3	1.1	8.5	0.9	10.8	0.5	9.2	1.6	9.1	1.3	9.0	0.8	8.0	1.1
Z	0.2	0.6	0.5	0.4	0.3	0.4	1.5	0.7	0.4	1.0	0.5	0.5	0.0	0.5	0.2	0.5
%	10.6	0.8	10.5	0.7	10.6	0.6	9.8	1.0	10.7	0.9	10.8	0.8	10.9	0.7	10.6	0.6
Residual mass																
kg	10.9	1.2	13.3	2.3	11.7	1.1	18.2	3.9	11.7	2.4	11.2	2.0	10.8	1.6	11.3	2.2
Z	3.2	1.1	3.7	1.0	3.2	0.9	6.4	2.5	3.6	1.7	3.7	1.4	3.0	1.3	3.2	1.3
%	13.0	0.9	12.9	0.8	12.7	0.6	14.3	1.2	13.6	1.2	13.3	1.0	12.9	1.3	12.8	0.9
Skin mass																
kg	4.1	0.2	4.6	0.4	4.4	0.2	5.1	0.4	4.2	0.2	4.1	0.3	4.2	0.2	4.3	0.3
%	5.0	0.2	4.6	0.3	4.7	0.2	4.0	0.3	4.9	0.3	4.9	0.3	5.0	0.2	4.9	0.3
Error																
kg	3.5	2.9	−1.7	0.9	0.5	1.6	2.0	2.5	4.2	4.1	3.5	4.7	3.7	3.5	−4.0	4.0
%	4.2	3.5	−1.5	1.0	0.5	1.7	1.6	1.8	4.8	4.1	4.2	5.3	4.4	4.3	−4.1	3.87
Somatotype																
Endomorphy	2.7	0.8	4.6	1.6	3.4	1.0	6.4	1.3	3.7	1.7	3.1	1.2	2.7	1.0	3.8	1.7
Mesomorphy	6.4	0.8	7.7	1.2	7.1	1.0	8.3	1.3	6.2	1.2	6.6	0.7	5.9	0.6	7.0	1.3
Ectomorphy	1.0	0.6	0.4	0.4	0.7	0.4	0.2	0.1	1.0	0.7	0.8	0.6	1.4	0.6	0.8	0.6
Indices																
BMI (kg/m^2^)	27.1	1.7	33.0	4.1	29.0	2.0	37.2	4.2	27.8	3.5	27.7	2.3	26.3	2.0	30.0	4.8
∑6SF (mm)	63.3	18.3	108.3	40.2	79.8	23.7	150.0	35.0	81.7	32.4	71.0	22.4	62.8	21.3	89.1	40.8
∑8SF (mm)	5.1	0.4	5.2	0.4	5.1	0.4	5.2	0.7	5.2	0.2	5.0	0.4	4.9	0.4	5.0	0.4
Arm span to Stretch stature	102.5	2.8	102.9	2.0	101.9	2.9	102.7	2.1	102.1	1.8	102.6	2.0	101.6	2.6	102.3	2.5
Cormic	52.6	1.3	52.5	1.2	52.4	1.7	52.5	1.2	52.2	1.6	52.8	1.4	52.4	1.1	52.1	1.5
Relative Biacromial Breadth	23.2	1.1	23.7	1.0	22.8	1.2	23.4	0.9	23.9	1.1	23.1	1.0	23.7	0.7	23.2	0.8
Relative Biiliocristal Breadth	16.1	0.8	17.4	1.1	16.9	1.4	16.6	1.0	18.5	0.9	16.5	1.5	16.3	0.8	16.0	0.9
Shoulder-Hip	69.6	5.1	73.5	5.2	70.8	5.3	77.7	4.9	71.8	4.7	68.9	3.8	69.2	3.9	71.8	5.6
Manouvrier	47.4	1.3	47.5	1.2	47.5	1.2	47.7	1.5	47.2	1.4	47.5	1.1	47.9	1.5	47.6	1.3
Muscle-to-bone ratio	5.1	0.4	5.2	0.4	5.1	0.4	5.2	0.7	5.2	0.2	5.0	0.4	4.9	0.4	5.0	0.4
Locomotive	0.53	0.07	0.61	1.0	0.56	0.07	0.68	0.07	0.62	0.09	0.55	0.06	0.54	0.07	0.58	0.09

DB: defensive backs; DL: defensive linemen; LB: linebackers; OL: offensive linemen; QB: quarterbacks; RB: running backs; WR: wide receivers; X: mean; SD: standard deviation; BMI: body mass index.

**Table 3:** Body composition and anthropometric indices in professional American football players in Mexico by playing position. Effect sizes (η^2^ or ε^2^) are reported for all variables. Post hoc comparisons were performed using the Games–Howell test for ANOVA variables and the Dunn test for Kruskal–Wallis variables.

	Sig.	Post Hoc	η^2^/ε^2^	Effect
Body mass (kg) ^b^	<0.05	OL>DL=LB=DB>QB, WR, RB	0.621	large
Stretch stature (cm) ^b^	<0.05	OL>DL>LB>DB>WR>RB>QB	0.297	large
Body composition				
Adipose mass				
kg ^a^	<0.05	OL>DL>LB>Rest	0.581	large
% ^b^	<0.05	OL>DL>LB=DB>Rest	0.297	large
Muscle mass				
kg ^b^	<0.05	OL>DL>LB>DB=Rest	0.545	large
% ^a^	<0.05	OL>DL>LB=DB>Rest	0.638	large
Skeletal mass				
kg ^b^	<0.05	OL>DL>LB>DB>Rest	0.638	large
% ^a^	<0.05	OL>DL>LB=DB>Rest	0.545	large
Residual mass				
kg ^b^	<0.05	OL>DL>LB>Rest	0.571	large
% ^b^	<0.05	OL>DL>LB>Rest	0.188	medium
Skin mass				
kg ^b^	<0.05	OL>DL>LB>Rest	0.595	large
% ^b^	<0.05	OL>DL>LB>DB>Rest	0.611	large
Indices				
Body mass index (kg/m^2^) ^b^	<0.05	OL>DL>LB>DB>Rest	0.577	large
∑6P (mm) ^b^	<0.05	OL>DL>LB>DB>Rest	0.453	large
Muscle to bone ratio ^a^	<0.05	ns	0.093	small
Arm span to Stretch stature ^a^	>0.05	ns	0.038	small
Cormic ^a^	>0.05	ns	0.020	small
Biacromial ^a^	>0.05	ns	0.078	small
Biiliocristal ^b^	<0.05	OL>DL>LB>QB>Rest	0.332	medium
Shoulder-Hip ^a^	<0.05	OL>DL>LB>QB>Rest	0.035	small
Manouvrier ^a^	>0.05	ns	0.020	small
Locomotive ^a^	<0.05	OL>QB>DL>Rest	0.309	small

^a^: One-way ANOVA (post hoc: Games–Howell); ^b^: Kruskal–Wallis (post hoc: Dunn); Sig. = *p* < 0.05 (significant differences); ns = no significant differences; DB: defensive backs; DL: defensive linemen; LB: linebackers; OL: offensive linemen; QB: quarterbacks; RB: running backs; WR: wide receivers.

## Data Availability

The original contributions presented in this study are included in the article. Further inquiries can be directed to the corresponding author.

## Abbreviations

The following abbreviations are used in this manuscript:

LFA	Professional American Football League in México
DF	Discriminant function
BMI	Body Mass Index
SAM	Somatotype Attitudinal Mean
DB	Defensive backs
DL	Defensive linemen
LB	Linebackers
OL	Offensive linemen
QB	Quarterbacks
RB	Running Backs
WR	Wide Receivers
